# Empirical Evaluation of Post Hoc Interpretability Methods for Clinical Trust in Deep Learning Diagnostics

**DOI:** 10.3390/diagnostics16203407

**Published:** 2026-10-20

**Authors:** K. R. Saranya, Y. Suganya

**Affiliations:** Department of Computer Science and Engineering, School of Computing, SRM Institute of Science and Technology, Tiruchirappalli 621105, India; suganyasuchithrra@gmail.com

**Keywords:** computational intelligence, explainable AI (XAI), deep learning, post-hoc interpretability, diagnostic support systems (DSS), SHAP, LIME, Grad-CAM

## Abstract

**Background/Objectives:** Brain tumor classification from magnetic resonance imaging (MRI) has been significantly improved through deep learning techniques; however, the lack of interpretability remains a major challenge for clinical adoption. **Methods:** This study proposes an explainable artificial intelligence (XAI)-driven framework for multiclass brain tumor classification using a baseline convolutional neural network (CNN) and four transfer learning models, namely ResNet50, DenseNet121, MobileNetV2, and EfficientNetB0. The models were trained and evaluated on a publicly available brain MRI dataset consisting of glioma, meningioma, and pituitary tumor classes. Their classification performance was assessed using accuracy, precision, recall, F1-score, receiver operating characteristic area under the curve (ROC-AUC), and confusion matrix analysis. To enhance transparency and interpretability, Grad-CAM, LIME, and SHAP were used to visualize and quantify the regions contributing to the classification decisions. **Results:** Experimental results state that the transfer learning models consistently outperformed the CNN by learning more discriminative feature representations while producing more localized and interpretable explanations. The agreement among Grad-CAM, LIME, and SHAP confirms that the proposed models predominantly relied on tumor regions rather than irrelevant background information, thereby improving the interpretability and reliability of the diagnostic predictions. **Conclusions:** The proposed framework provides an effective and explainable computer-aided diagnosis system that supports trustworthy deep learning-based brain tumor classification and has the potential for decision-making.

## 1. Introduction

Deep learning (DL) has emerged as one of the most important technologies in modern healthcare by providing intelligent diagnostic support systems capable of analyzing complex medical images, physiological signals, and electronic health records with remarkable accuracy [1]. Recent advances in convolutional neural networks (CNNs) and transfer learning architectures have noticeably improved the automatic detection and classification of various diseases, thereby reducing diagnostic time and assisting clinicians in making informed decisions. In medical image analysis, deep learning models have demonstrated promising performance in identifying subtle pathological patterns that may not be easily recognized through conventional image processing techniques [2]. Despite these remarkable achievements, widespread adoption of the deep learning models in clinical practice remains limited due to their underlying “black-box” nature. Although these models can achieve high predictive accuracy, they often fail to provide transparent explanations for their predictions, making it difficult for clinicians to understand, verify, and trust the decision-making process. Since medical diagnosis directly affects patient care and treatment planning, transparency, reliability, and accountability have become essential requirements for deploying artificial intelligence (AI)-based diagnostic systems in healthcare environments [3].

To improve model transparency, several post-hoc explainability techniques have been developed to interpret the decisions of trained deep learning models. Among these, Gradient-weighted Class Activation Mapping (Grad-CAM), Local Interpretable Model-Agnostic Explanations (LIME), and SHapley Additive exPlanations (SHAP) are widely adopted for visualizing and explaining model predictions [4,5]. These methods allow researchers and clinicians to identify image regions or features that contribute most significantly to the model’s prediction, thereby better understanding the model’s decision-making process. However, the effectiveness of these explainability techniques may vary depending on the underlying deep learning architecture and the characteristics of the medical imaging dataset [6].

Although numerous studies have reported high diagnostic performance using deep learning techniques, many investigations focus on a single CNN architecture or evaluate only one explainability method [7]. Consequently, there is limited understanding of how different deep learning architectures perform under identical experimental conditions and how their predictions can be effectively interpreted using multiple explainability approaches. Furthermore, the comprehensive comparative studies that simultaneously evaluate predictive performance and interpretability using standardized evaluation metrics remain relatively limited in the existing literature [8].

To address these research gaps, this study presents a comprehensive comparative evaluation of the Convolutional Neural Network (CNN) and four widely adopted transfer learning architectures, namely ResNet50, DenseNet121, EfficientNetB0, and MobileNetV2, for medical image classification [9]. The proposed framework evaluates the predictive performance of these models using multiple quantitative metrics, including accuracy, precision, recall, F1-score, Receiver Operating Characteristic–Area Under the Curve (ROC-AUC), and the confusion matrix analysis. Furthermore, the three widely used post-hoc explainability methods, namely Grad-CAM, SHAP, and LIME, are employed to interpret the predictions of the trained models and analyze their ability to provide meaningful visual explanations [10].

The major contributions of this study are summarized as follows:

Development of a comparative framework incorporating the CNN and four widely adopted transfer learning architectures for medical image classification. Systematic evaluation of model performance using multiple quantitative evaluation metrics under a unified experimental protocol. The goal of comparing Grad-CAM, SHAP, and LIME is to see how well they can explain deep learning predictions [7,11]. This analysis examines the trade-off between predictive performance and model interpretability to support the development of transparent and reliable AI-assisted diagnostic systems.

**Figure 1:**
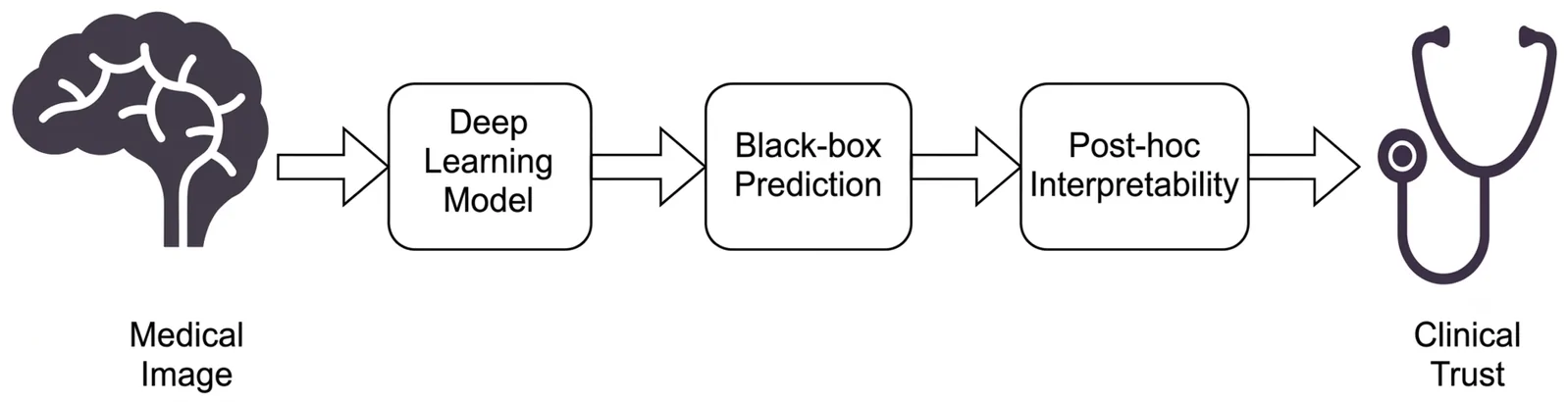
Workflow of Explainable Deep Learning Method.

The organization of the paper is as follows. Section 2 reviews the related literature on deep learning-based medical image diagnosis and explainable artificial intelligence techniques. Section 3 presents the proposed methodology and the experimental framework. Section 4 describes the results and discussion. Section 5 concludes the paper by summarizing the major findings, limitations, and future research directions. The workflow of the deep learning model is given in Figure 1.

## 2. Literature Review

Deep learning has revolutionized medical image diagnosis by enabling automatic extraction of meaningful features directly from the medical images without relying on handcrafted feature engineering. These models have demonstrated remarkable capability in identifying, classifying, segmenting, and monitoring various diseases with the help of imaging modalities such as magnetic resonance imaging (MRI), computed tomography (CT), X-ray, ultrasound, retinal fundus imaging, etc. Their ability to learn complex hierarchical representations has significantly improved the efficiency and reliability of computer-aided diagnostic systems [12].

Among the various deep learning approaches, Convolutional Neural Networks (CNNs) have become the basis of modern medical image analysis. CNNs automatically extract the spatial features through convolutional operations, enabling the accurate identification of disease-specific patterns from medical images. Unlike traditional machine learning approaches, which require manual feature extraction, CNNs learn discriminative features directly from raw image data through multiple convolutional and pooling layers. This capability has enabled the CNN-based models to achieve a high diagnostic performance across numerous clinical applications, including brain tumor detection, diabetic retinopathy screening, skin lesion classification, breast cancer diagnosis, pneumonia detection, and neurological disorder prediction [13].

Although conventional CNNs have achieved promising diagnostic accuracy, increasing the depth of neural networks often introduces optimization challenges such as vanishing gradients, degradation of training performance, and overfitting. To overcome these limitations, several advanced deep learning architectures have been developed. Residual learning introduces shortcut connections that allow information to bypass the intermediate layers, enabling the successful training of deeper neural networks. Dense connectivity further improves feature propagation by connecting each layer with every subsequent layer, encouraging feature reuse and strengthening the gradient flow throughout the network. These architectural improvements have significantly improved the feature representation while reducing the information loss during training.

Recent advancements have also focused on designing computationally efficient architectures suitable for real-world clinical deployment. Lightweight deep learning models reduce the memory requirements and computational complexity without significantly compromising predictive performance. Efficient scaling strategies optimize network depth, width, and image resolution simultaneously, whereas depthwise separable convolutions minimize the computational cost while preserving feature extraction capability. Such lightweight architectures have become increasingly important for mobile healthcare applications, portable diagnostic devices, telemedicine platforms, and edge computing environments where computational resources are limited.

Transfer learning has emerged as an effective strategy for addressing the limited availability of annotated medical datasets. Instead of training deep neural networks from scratch, transfer learning utilizes knowledge acquired from large-scale image datasets and adapts the learned representations to specific medical imaging tasks. Fine-tuning pre-trained models accelerates convergence, reduces computational requirements and improves generalization performance, which minimizes overfitting. This approach has become particularly valuable in medical image analysis because obtaining expert-annotated datasets is often expensive, time-consuming, and subject to strict privacy regulations [14].

Despite these remarkable advancements, several challenges continue to limit the widespread clinical adoption of deep learning-based diagnostic systems. Most studies primarily focus on improving predictive accuracy while paying comparatively less attention to model transparency and interpretability. Furthermore, differences in datasets, preprocessing techniques, experimental protocols, and the evaluation metrics make direct comparisons among different deep learning architectures difficult. These inconsistencies reduce the reproducibility of experimental findings and complicate the identification of the most suitable model for practical deployment.

In general, deep learning methods have significantly improved medical image diagnosis by improving disease detection accuracy, reducing diagnostic time, and supporting clinicians in decision-making. However, as deep learning architectures become increasingly complex, understanding the reasoning behind their predictions has become equally important. The growing demand for transparent, interpretable, and trustworthy diagnostic systems has, therefore, driven the development of Explainable Artificial Intelligence (XAI) techniques, which aim to provide meaningful explanations for deep learning predictions [15]. The following section focuses on the main explainability methods employed to improve the transparency of deep learning-based diagnostic systems.

### 2.1. Explainable Artificial Intelligence in Medical Imaging

Deep learning models have achieved significant results in medical image analysis; however, their complex architecture often limits the transparency of the decision-making process. The inability to explain how a model arrives at a particular prediction reduces clinician confidence and presents challenges for the safe adoption of artificial intelligence in healthcare. Consequently, Explainable Artificial Intelligence (XAI) has emerged as an important research area that aims to enhance the interpretability, transparency, and trustworthiness of deep learning models by providing meaningful explanations for their predictions [16].

XAI techniques are broadly categorized into intrinsic and post-hoc approaches. Intrinsic methods employ inherently interpretable models, whereas the post-hoc methods generate explanations after model training without modifying the underlying architecture. Considering their flexibility and compatibility with existing deep learning models, post-hoc explainability techniques have become the preferred choice for medical image analysis [17]. These methods assist clinicians in understanding whether the model focuses on relevant image regions and features before accepting its predictions.

Among the most available post-hoc techniques, Gradient-weighted Class Activation Mapping (Grad-CAM), Local Interpretable Model-Agnostic Explanations (LIME), and SHapley Additive exPlanations (SHAP) are widely adopted. Grad-CAM produces class-specific heatmaps that highlight important image regions responsible for the prediction, making it particularly suitable for convolutional neural networks. LIME explains individual predictions by approximating the complex model with a simpler interpretable model in the local neighborhood of the input sample, whereas SHAP estimates the contribution of each feature using concepts from cooperative game theory, providing both local and global model interpretations [18].

Although these explainability methods have significantly improved the transparency of deep learning models, each possesses distinct advantages and its own limitations. Grad-CAM provides intuitive visual localization but is mainly applicable to CNN-based architectures. LIME offers model-agnostic local explanations but may produce inconsistent results due to its sampling strategy. SHAP provides theoretically consistent feature importance estimates, but its computational complexity can be high for large deep learning models. Therefore, comparative evaluation of these explainability techniques is essential for identifying the most suitable approach for reliable decision-making.

The following section presents a comparative summary of recent studies on deep learning and explainable artificial intelligence in medical imaging, highlighting their major contributions, limitations, and the research gaps that motivate the present work. Table 1 provides an overview of the existing work on post-hoc interpretability techniques used in deep learning-based diagnostic systems. This section helps to understand the current research in the field and the research gap that motivates the need for the comparative evaluation.

### 2.2. Critical Analysis and Research Gap

The existing literature explains deep learning techniques that have significantly improved medical image diagnosis by achieving high predictive performance across various clinical applications. Meanwhile, Explainable Artificial Intelligence (XAI) techniques such as Grad-CAM, LIME, and SHAP have enhanced model transparency by providing visual and feature-based explanations that improve clinician confidence. However, most existing studies focus on a single deep learning architecture or a single explainability technique, limiting comprehensive evaluation of diagnostic performance and interpretability.

Further, the variations in datasets, preprocessing methods, experimental protocols, and evaluation metrics make direct comparison among existing studies difficult. Several studies emphasize predictive accuracy while providing only qualitative assessments of explanations, with limited analysis of explanation reliability and computational efficiency. These limitations reduce the reproducibility of results and make it challenging to identify the most suitable deep learning and XAI combination for real-world healthcare applications.

Based on these observations, a clear research gap exists in the lack of a unified comparative framework that evaluates multiple deep learning architectures together with widely adopted post-hoc explainability methods under identical experimental conditions. To address this gap, the present study performs a comparative evaluation of the CNN and four transfer learning models, ResNet50, DenseNet121, EfficientNetB0, and MobileNetV2, using consistent datasets and evaluation metrics [30]. In addition, Grad-CAM, LIME, and SHAP are applied to analyze the model interpretability, providing a comprehensive assessment of both predictive performance and explainability for AI-interpreted medical diagnosis.

## 3. Methodology

**Figure 2:**
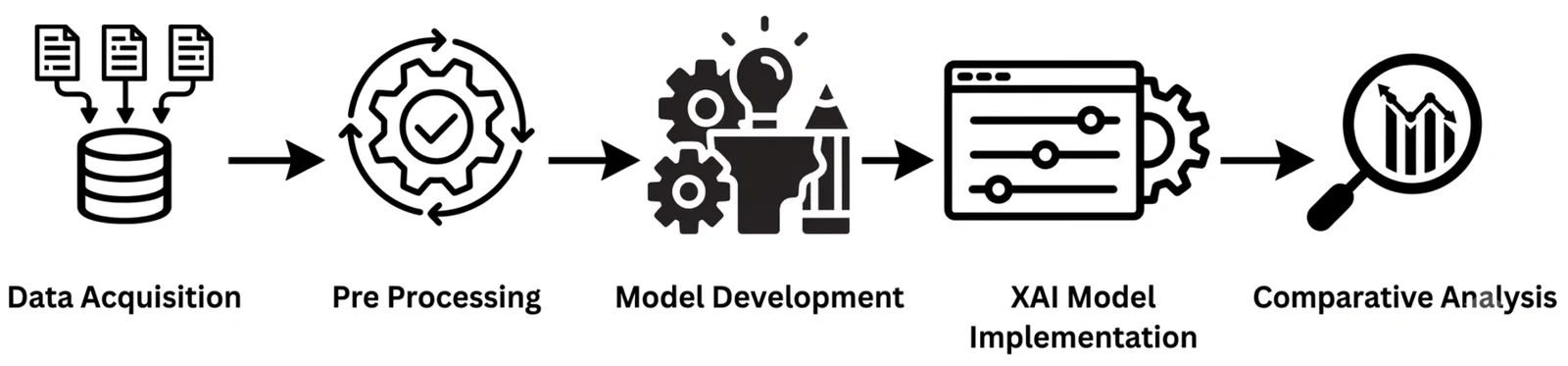
Overall workflow of the proposed framework for brain tumor analysis.

This study performs a comparative evaluation of a baseline Convolutional Neural Network (CNN) and four widely adopted transfer learning architectures, namely ResNet50, DenseNet121, EfficientNetB0, and MobileNetV2, for medical image classification [30]. All the models receive preprocessed medical images of identical dimensions and are trained under the same experimental settings to ensure a fair comparison. For the transfer learning models, ImageNet pre-trained weights are used, and the original classification layers are replaced with the task-specific fully connected layers corresponding to the target classes. The following subsections briefly explain the architecture of each model. The overall workflow of the proposed system is illustrated in Figure 2.

### 3.1. Dataset Description

**Figure 3:**
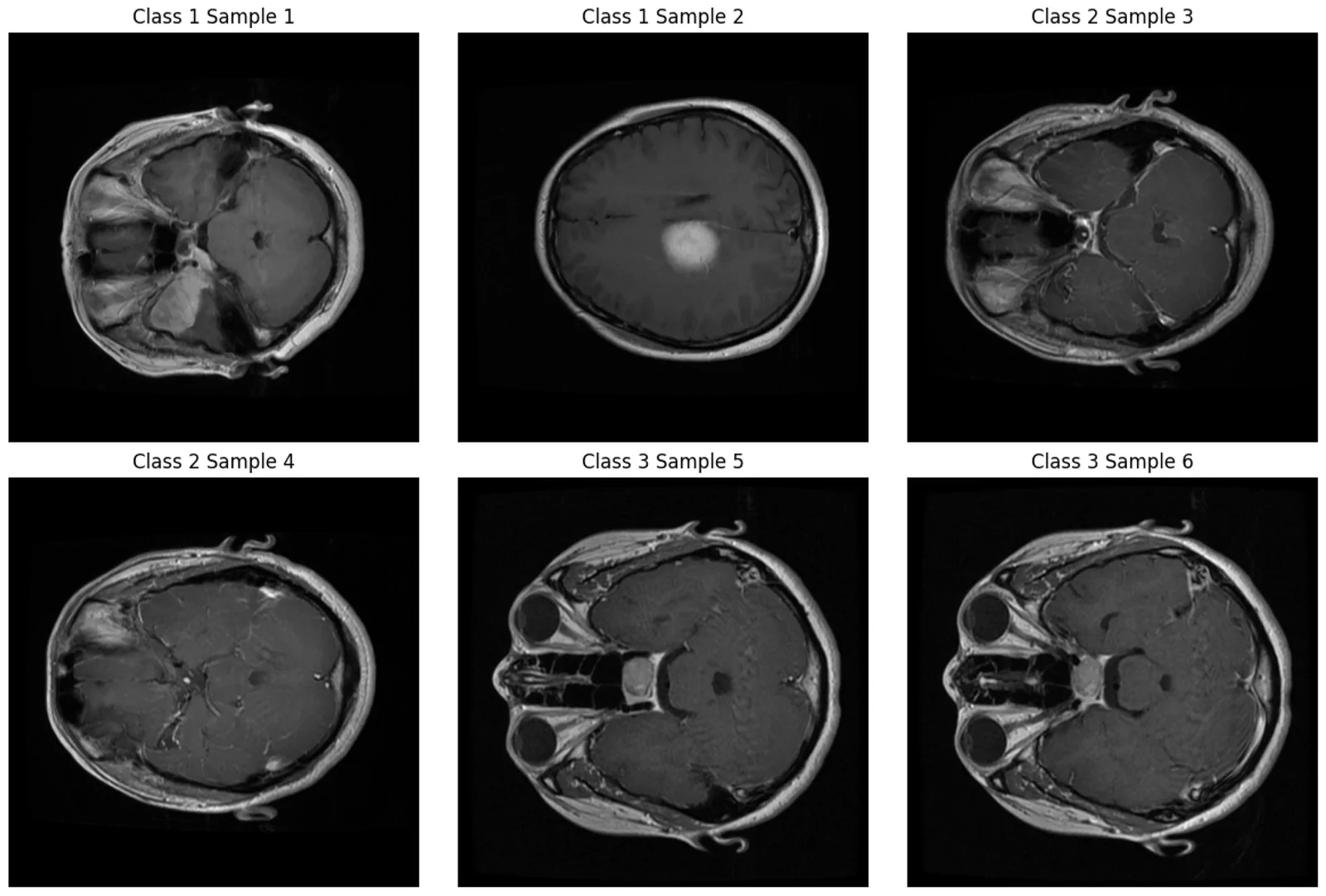
Brain MRI samples (Figshare Brain Tumor MRI dataset).

The study is performed using the Figshare Brain Tumor MRI Dataset, publicly available on Kaggle. The dataset consists of T1-weighted MRI brain scans of clinically diagnosed patients. The dataset consists of three tumor classes: glioma, meningioma, and pituitary tumor. The dataset also contains 2D MRI slices derived from volumetric scans, which helps to reduce the computational and storage requirements compared with 3D MRI datasets. All images are provided in standard image formats and exhibit variations in resolution, intensity, and anatomical orientation, reflecting real-world clinical heterogeneity [31]. To make sure about the compatibility of the deep learning models, the MRI images were resized to a fixed resolution, also the dataset was divided into training, validation and testing subset. This dataset was selected due to its widespread use in deep learning-based brain tumor classification studies and its ability to support meaningful evaluation of interpretability methods aimed at enhancing trust in diagnostic support systems. Figure 3 illustrates the brain MRI samples from the Figshare Brain Tumor MRI dataset. Each row represents the axial slices from three tumor classes: Class 1—Meningioma: it is typically a benign tumor generated from the meninges, the protective layers surrounding the brain and spinal cord. Class 2—Glioma: Malignant tumors originating from the glial cells. Class 3—Pituitary Tumor: it is usually a benign adenoma located in the pituitary gland, which can disrupt hormonal balance or compress nearby structures.

### 3.2. Data Preprocessing

All the images present in the Brain MRI samples (Figshare Brain Tumor MRI dataset) were originally distributed in MATLAB v7.3. MAT format. They were accessed using the h5py library to ensure compatibility with HDF5-based storage. All the images were extracted from the cjdata structure and converted into NumPy arrays for further preprocessing. To maintain the standard pixel intensity, all the images were normalized to the range 0 to 1, which improves model convergence. The original image varied in resolution, so each image was resized to a fixed dimension of 128 × 128 pixels. Finally, the tumor class labels (originally encoded as integers 1, 2, and 3) were flattened and transformed into a one-hot encoded representation with three output categories, enabling the convolutional neural network to perform multiclass classification of Meningioma, Glioma, and Pituitary Tumor cases.

### 3.3. Model Architecture

#### 3.3.1. Convolutional Neural Network (CNN)

**Figure 4:**
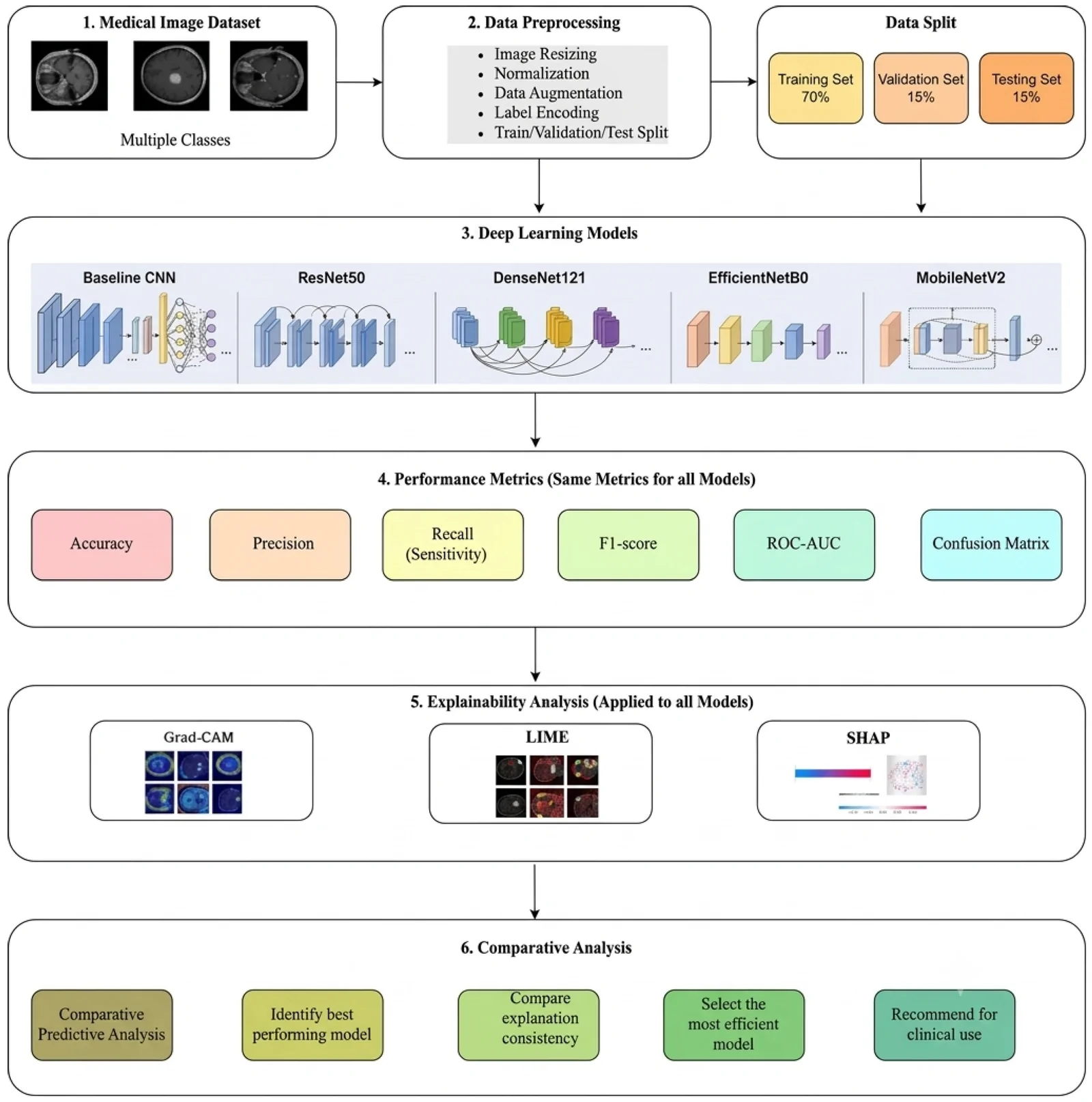
Overall framework of the comparative study for medical image classification and explainability.

CNN is designed to provide a reference for evaluating the effectiveness of advanced transfer learning architectures. The network includes multiple convolutional layers for automatic feature extraction, followed by max-pooling layers that reduce spatial dimensions while preserving important image characteristics. Batch normalization and dropout layers are used to improve training stability and reduce overfitting. At the end, fully connected layers and a Softmax classifier are applied to classify the input images into their respective categories. The CNN serves as the base to which the performance of the pre-trained deep learning architectures is compared [32]. Figure 4 represents the overall framework of the comparative study for medical image classification and explainability.

#### 3.3.2. ResNet50

ResNet50 is a 50-layer deep convolutional neural network that makes use of residual learning through shortcut connections. These residual connections allow the information to bypass the intermediate layers, effectively addressing the vanishing gradient problem and enabling the training of deeper networks. The architecture has demonstrated excellent performance in various computer vision and medical imaging applications by understanding rich hierarchical feature representations. In this study, the ImageNet pre-trained ResNet50 model is fine-tuned for the medical image classification task [33].

#### 3.3.3. EfficientNetB0

EfficientNetB0 employs a compound scaling strategy that simultaneously scales network depth, width, and input resolution using fixed scaling coefficients. This balanced scaling approach enables EfficientNetB0 to achieve competitive classification accuracy with relatively fewer parameters and lower computational complexity. Due to its favorable balance between efficiency and predictive performance, EfficientNetB0 has become a popular architecture for medical image classification and is evaluated as one of the representative transfer learning models in this study [34].

#### 3.3.4. MobileNetV2

MobileNetV2 is a lightweight convolutional neural network designed for computationally efficient image classification. The architecture makes use of depthwise separable convolutions and inverted residual blocks with linear bottlenecks to reduce the computational cost while preserving feature extraction capability. These characteristics make MobileNetV2 more suitable for deployment in resource-constrained environments such as mobile devices and embedded healthcare systems. In this work, MobileNetV2 is incorporated to evaluate whether lightweight architectures can achieve competitive diagnostic performance while maintaining computational efficiency [35].

### 3.4. Model Training

All deep learning models were trained under the identical experimental settings to ensure a reliable and unbiased comparative evaluation. The CNN, ResNet50, DenseNet121, EfficientNetB0, and MobileNetV2 models were deployed using the TensorFlow–Keras framework. The Adam optimizer was used because of its adaptive learning rate mechanism and the efficient convergence characteristics for deep learning optimization. The categorical cross-entropy loss function was used, as the task involved multiclass classification of brain tumor images into Meningioma, Glioma, and Pituitary Tumor classes.

The dataset was trained using a batch size of 32, while the number of training epochs varied depending on the architecture and the convergence behavior of each model. Since the aim is to improve the training process and prevent overfitting, Early Stopping and ReduceLROnPlateau callbacks were deployed. Early Stopping helped to check the validation loss and stopped the training when no further improvement was measured, whereas ReduceLROnPlateau automatically reduced the learning rate based on the performance stagnation. Model checkpoints were also utilized to preserve the best-performing weights during training.

The predictive performance of all the models was evaluated using a common set of quantitative metrics, which includes accuracy, precision, recall, F1-score, Receiver Operating Characteristic–Area Under the Curve (ROC–AUC), and confusion matrix analysis. Following the identical preprocessing steps, training parameters, and evaluation metrics across all the models ensured a reliable and objective comparison of predictive performance and interpretability. The training parameters used for all the deep learning models are given in Table 2.

### 3.5. Interpretability Methods

To enhance the transparency of deep learning predictions, three widely adopted post-hoc explainability techniques, namely Gradient-weighted Class Activation Mapping (Grad-CAM), Local Interpretable Model-Agnostic Explanations (LIME), and SHapley Additive exPlanations (SHAP) [36], were used in this study. These methods were applied to the trained deep learning models to generate the visual and feature-level explanations of their predictions. Grad-CAM highlights the image regions that contribute most significantly to the predicted class, LIME focuses on the individual predictions through local surrogate models, and SHAP quantifies feature contributions using Shapley values derived from cooperative game theory. Altogether, these complementary techniques enable a comprehensive assessment of model interpretability and facilitate comparison of explanation quality across the evaluated deep learning architectures.

## 4. Results and Discussion

### 4.1. Comparative Performance Evaluation

To evaluate the predictive capability of the proposed framework, the Convolutional Neural Network (CNN) and four transfer learning architectures, namely ResNet50, DenseNet121, EfficientNetB0, and MobileNetV2, were trained and evaluated under the identical experimental conditions. All models were evaluated using the same training, validation, and testing datasets to ensure a consistent and unbiased comparison. The predictive performance was measured using accuracy, precision, recall, F1-score, and Receiver Operating Characteristic–Area Under the Curve (ROC-AUC). Table 3 represents the comparative performance of CNN and transfer learning models.

### 4.2. Confusion Matrix Analysis

**Figure 5:**
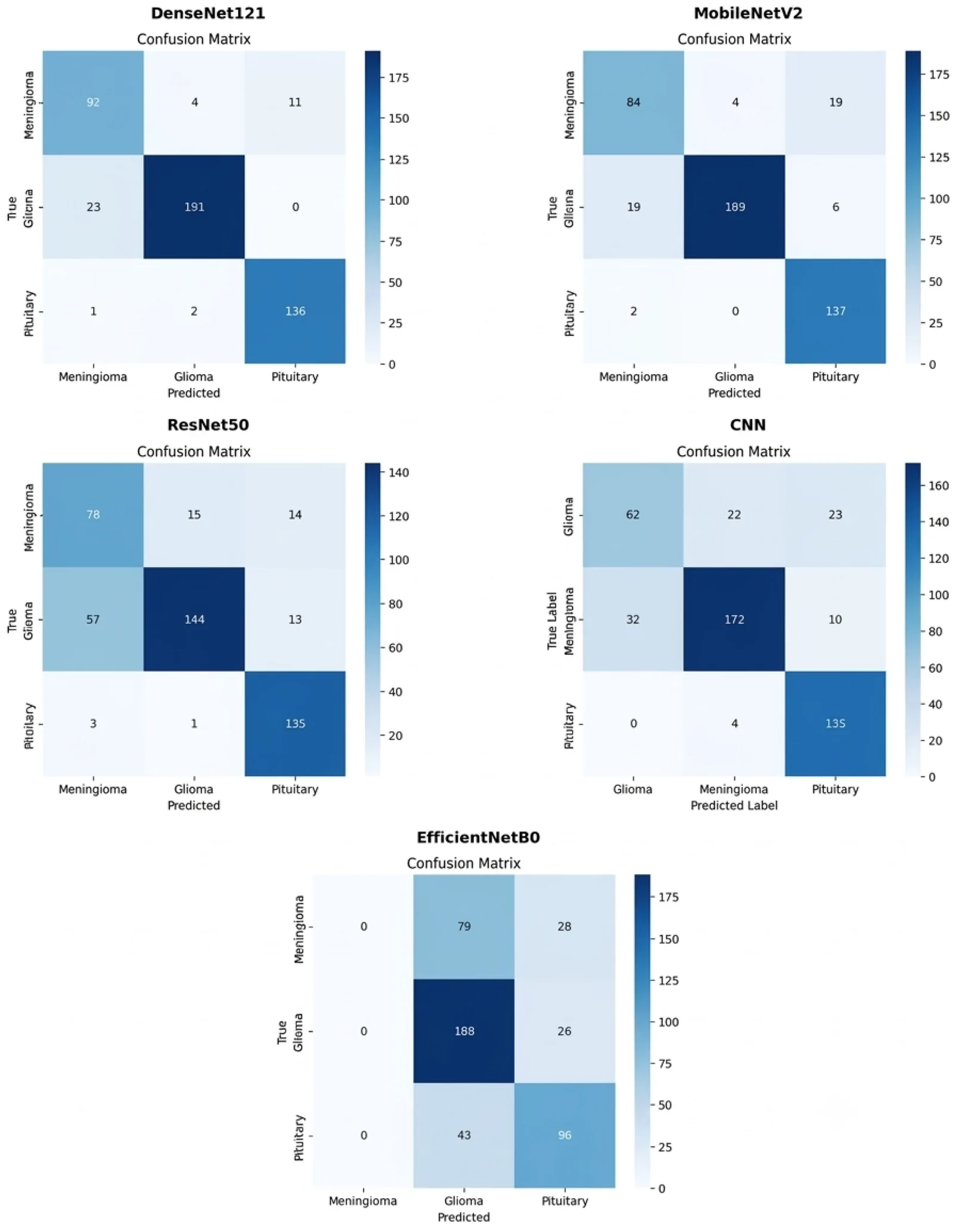
Confusion matrices of the evaluated deep learning models for brain tumor classification.

The confusion matrices of the CNN and the four transfer learning models are presented in Figure 5 to understand the class-wise evaluation of the classification performance. Among the evaluated models, DenseNet121 achieved the highest number of correctly classified samples with minimal confusion between the brain tumor classes. MobileNetV2 achieved strong classification results with relatively few misclassifications, whereas the CNN and ResNet50 showed moderate performance with a higher number of classification errors. EfficientNetB0 produced a large number of misclassified samples. Overall, the confusion matrix analysis helps to understand the quantitative evaluation results and validates that DenseNet121 provides the most reliable and strong classification performance for the brain tumor dataset.

### 4.3. Explainable Artificial Intelligence (XAI) Analysis

#### 4.3.1. Explainability Analysis of the CNN Model

**Figure 6:**
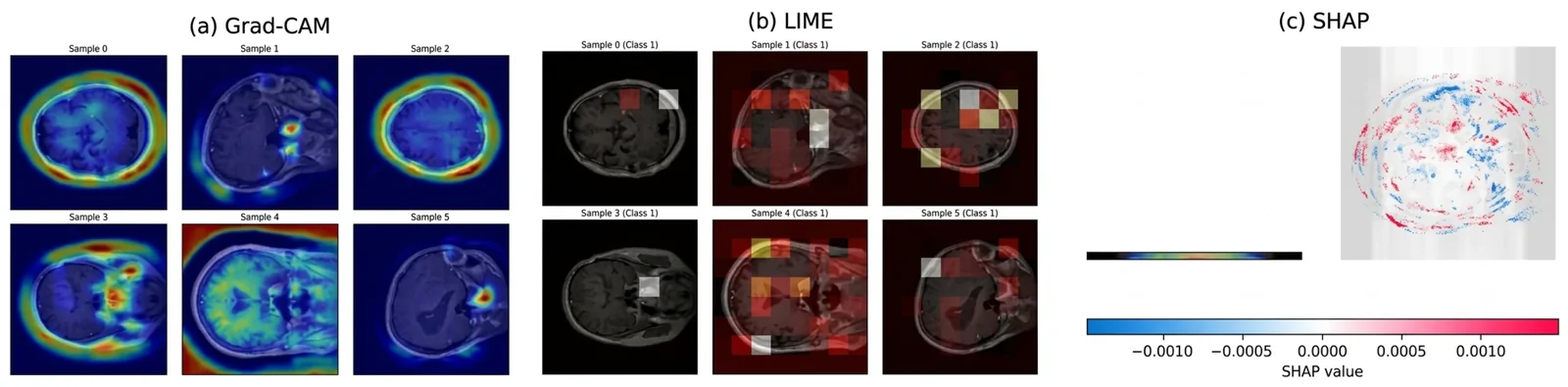
Explainability analysis of the baseline CNN model using three methods: (**a**) Grad-CAM, (**b**) LIME, and (**c**) SHAP.

To improve the interpretability of the CNN model, Grad-CAM, LIME, and SHAP were used to visualize the areas influencing the classification decisions. Grad-CAM highlighted the tumor areas that are responsible for the predictions; meanwhile, LIME found the local image superpixels that contributed most predominantly to the predicted class. The SHAP explanation further helped to identify the positive and negative contributions of different image regions, indicating the model primarily relied on clinically relevant tumor features. The consistent observations among the three explainability methods indicate that the CNN focused primarily on the tumor region during classification, thereby enhancing the transparency and reliability of its predictions. Figure 6 illustrates the explainability analysis of the CNN model using Grad-CAM, LIME, and SHAP.

#### 4.3.2. Explainability Analysis of Transfer Learning Models

To further investigate the interpretability of the proposed transfer learning models, Grad-CAM, LIME, and SHAP were applied to ResNet50, DenseNet121, MobileNetV2, and EfficientNetB0 using the same MRI image. This consistent evaluation strategy enables a direct comparison of the attention mechanisms and feature attribution characteristics of different deep learning architectures.

##### Grad-CAM Analysis

**Figure 7:**
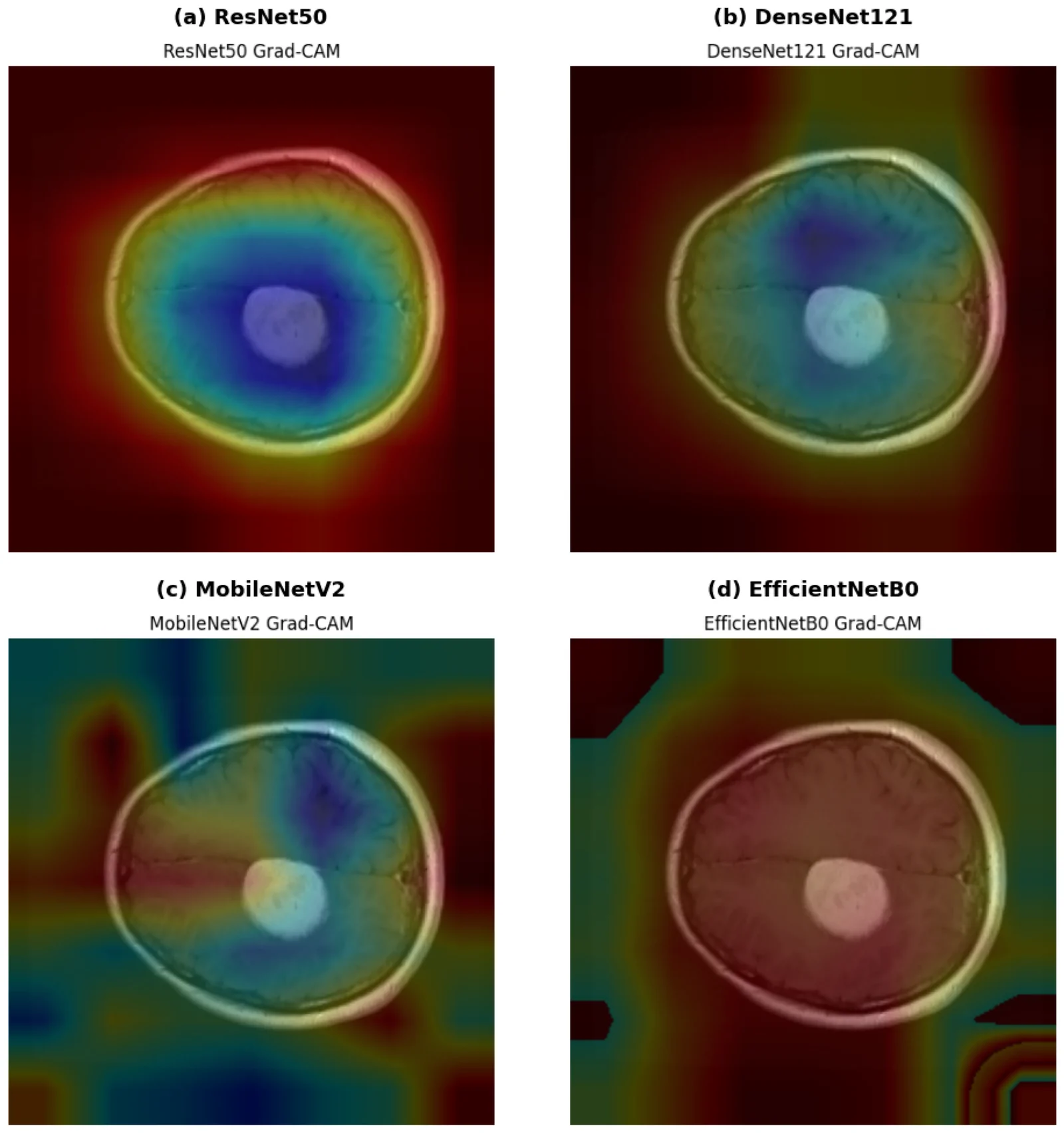
Comparison of Grad-CAM visualizations for the transfer learning models: (**a**) ResNet50, (**b**) DenseNet121, (**c**) MobileNetV2, and (**d**) EfficientNetB0.

The Grad-CAM visualizations revealed that all transfer learning models predominantly focused on the tumor region while generating predictions. Compared with the CNN, the activation maps produced by the transfer learning architectures were more localized and exhibited fewer activations over normal brain tissues. This indicates that the pre-trained networks learned more discriminative and clinically relevant representations. Among the evaluated models, ResNet50 and DenseNet121 achieved comparatively smoother and more localized activation maps, meanwhile MobileNetV2 and EfficientNetB0 produced relatively broader activation patterns around the tumor region. Figure 7 represents the comparison of Grad-CAM visualizations for transfer learning models.

##### LIME Analysis

**Figure 8:**
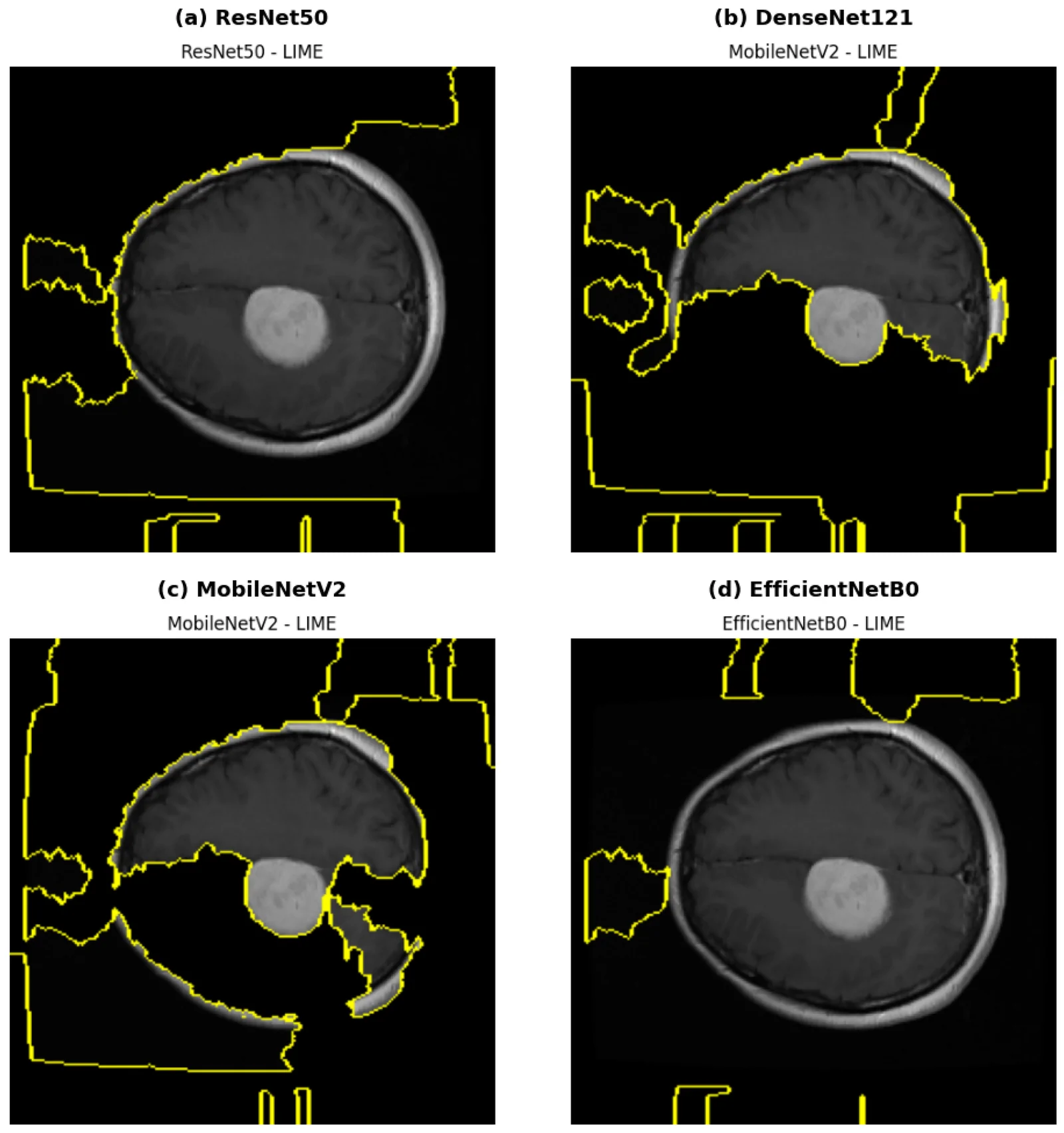
Comparison of LIME visualizations for the transfer learning models: (**a**) ResNet50, (**b**) DenseNet121, (**c**) MobileNetV2, and (**d**) EfficientNetB0.

The LIME explanations identified the image superpixels that contributed most strongly to each prediction. For all transfer learning models, the positively contributing regions were concentrated around the tumor mass, while comparatively fewer irrelevant regions were highlighted. This consistency indicates that the models depend primarily on pathological structures rather than the surrounding healthy tissues during classification. The localized explanations generated by LIME were highly consistent with the Grad-CAM activation maps, providing complementary evidence regarding the robustness of the learned representations. Figure 8 represents the comparison of LIME visualizations for transfer learning models.

##### SHAP Analysis

**Figure 9:**
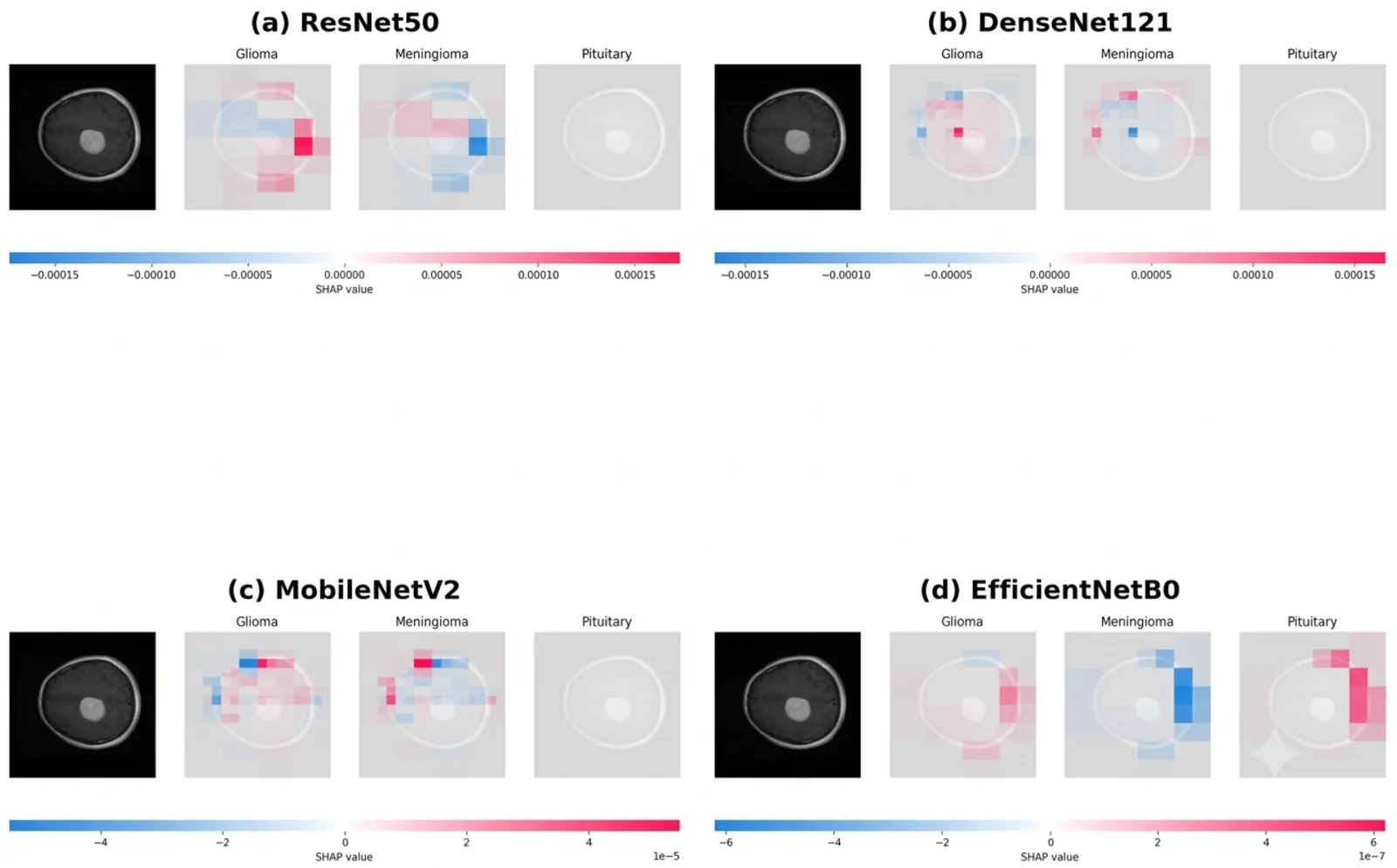
Comparison of SHAP visualizations for the transfer learning models: (**a**) ResNet50, (**b**) DenseNet121, (**c**) MobileNetV2, and (**d**) EfficientNetB0.

SHAP was used to quantify the contribution of different image regions toward each predicted tumor class. Positive SHAP values (red-indicated regions) increased the confidence of the predicted class, whereas negative SHAP values (blue-indicated regions) reduced the probability of competing classes. Across all four transfer learning models, the highest positive contributions consistently represent the tumor region, while normal brain tissues exhibited comparatively lower attribution values. The SHAP explanations closely aligned with both the Grad-CAM and LIME results, explaining the strong agreement among the three explainability methods. Such consistency improves the interpretability of the proposed framework and provides additional confidence that the models base their predictions on clinically meaningful imaging biomarkers rather than the spurious image characteristics. Figure 9 represents the comparison of SHAP visualizations for transfer learning models.

## 5. Conclusions

This study presented an explainable deep learning framework for multiclass brain tumor classification using the CNN and four transfer learning architectures, namely ResNet50, DenseNet121, MobileNetV2, and EfficientNetB0. Comparative performance evaluation demonstrated that the transfer learning models achieved superior classification performance over the CNN by learning more discriminative feature representations from brain MRI images. The incorporation of Grad-CAM, LIME, and SHAP enabled comprehensive interpretation of the model predictions by highlighting clinically relevant tumor regions and quantifying feature contributions. These findings help to show that combining transfer learning with complementary XAI techniques can improve both predictive performance and interpretability, making the framework more applicable for decision-support applications. Future work will focus on testing the proposed framework using multi-institutional datasets, incorporating multimodal MRI sequences, and evaluating its robustness in real-world clinical environments to further improve the generalizability and practical application of the explainable brain tumor diagnosis.

## Figures and Tables

**Table 1:** Literature Review Summary.

Ref.	Author and Year	Title of the Paper	Dataset Used	XAI Methods	Key Findings	Research Gap
[19]	Akira Sakai et al. (2022)	Medical Professional Enhancement Using Explainable Artificial Intelligence in Fetal Cardiac Ultrasound Screening	Fetal cardiac ultrasound scans	Graph chart diagram	Enhances fetal cardiac ultrasound screening accuracy	Lack of evaluation based on comparative study
[20]	Dost Muhammad et al. (2025)	Randomized Explainable Machine Learning Models for Efficient Medical Diagnosis	Genitourinary Cancers Dataset, Coronary Artery Disease Dataset	LIME, SHAP	Demonstrate the superiority of randomized machine learning models over traditional deep learning architectures	Extensive training duration and substantial resource requirements (both computational and data)
[21]	Tirtha Chanda et al. (2024)	Dermatologist-like explainable AI enhances trust and confidence in diagnosing melanoma	HAM10000 dataset (biopsy-verified melanoma and nevus images)	Grad-CAM and LRP	Diagnostic Accuracy (81% balanced accuracy)	Limited Evaluation of XAI Impact
[22]	Fatima Hasan Saif et al. (2024)	Explainable AI for Alzheimer Detection: A Review of Current Methods and Applications	ADNI (Alzheimer’s Disease Neuroimaging Initiative), OASIS (Open Access Series of Imaging Studies), UK Biobank	SHAP, LIME, Grad-CAM	AI models, especially Deep Learning (DL) techniques like CNNs, have shown high accuracy in detecting AD	Lack of True Interpretability
[23]	Panagiotis Papadimitroulas et al. (2021)	Artificial intelligence: Deep learning in oncological radiomics and challenges of interpretability and data harmonization	Small, retrospective and monocenter cohorts of patients	Proxy Models and Model Compression, Visualization of Intermediate Features, Importance Estimators and Relevance Scores	CNN achieved state-of-the-art performance in medical image	Lack of Interpretability and Explainability
[24]	Samanta Knapič et al. (2021)	Explainable AI for Human Decision Support System in Medical Domain	Red Lesion Endoscopy dataset	LIME, SHAP and CIU	CIU explainable method performed better than both LIME and SHAP	Improving the comprehensibility of decisions made by machine learning algorithms
[25]	Yassir Farooqui et al. (2025)	Explainable Fuzzy Logic in AI: Enhancing Transparency and Trust in Deep Learning Models	UCI Credit Risk Dataset, MIMIC-III Health Data, MNIST Handwritten Digits, KDD Cup 1999	Fuzzy Feature Engineering, Fuzzy Activation Functions, Fuzzy Decision Layers, Fuzzy Rule-Based Learning	The fuzzy logic-enhanced deep learning model achieved slightly lower accuracy, but with significantly improved explainability	Limited work on seamlessly integrating fuzzy logic into deep learning architectures
[26]	Kenji Karako and Wei Tang (2024)	Applications of and issues with machine learning in medicine: Bridging the gap with explainable AI	Electronic health record data	SHAP, LIME	Machine learning models have been effectively used to predict postoperative complications	Internal workings and decision-making processes of these models are opaque and difficult to interpret
[27]	Dost Muhammad and Malika Bendechache (2024)	Unveiling the black box: A systematic review of Explainable Artificial Intelligence in medical image analysis	ChestX-ray14, ISIC Skin Lesion Dataset, LUNA16, BraTS (Brain Tumor Segmentation) Dataset, MIMIC-CXR	Grad-CAM, Guided Grad-CAM, SHAP, LIME	XAI enhances trust and interpretability in medical image analysis	Developing standardized, robust, and interpretable explanation methods for medical image analysis to improve integration and validation
[28]	Fahad Ahmed et al. (2026)	Explainable artificial intelligence (XAI) in medical imaging: a systematic review of techniques, applications, and challenges	Chest X-ray Datasets, MRI Datasets, Histopathology Dataset, Ultrasound Datasets	Grad-CAM, SHAP, LIME	XAI methods enhance interpretability in medical imaging; challenges remain in ensuring reliability, consistency, and widespread adoption	Developing standardized, reliable, and useful XAI methods that address variability, validation, and integration challenges in medical imaging
[29]	Essam H. Houssein et al. (2025)	Explainable artificial intelligence for medical imaging systems using deep learning: a comprehensive review	Chest X-ray Datasets, Brain MRI Datasets, Cardiac Imaging Datasets, Dermatology Imaging Datasets, Lung and Pulmonary Datasets	Saliency Maps, Grad-CAM, LIME, SHAP, Attention Maps	XAI methods like Grad-CAM, saliency maps, and integrated gradients enhance interpretability and trustworthiness of deep learning models in medical imaging	Limited validation of XAI methods across diverse medical imaging modalities, and disease diagnoses to ensure their robustness and generalizability

**Table 2:** Training parameters used for all deep learning models.

Parameter	Value
Framework	TensorFlow–Keras
Optimizer	Adam
Loss Function	Categorical Cross-Entropy
Batch Size	32
Input Image Size	224×224 pixels
Data Augmentation	Rotation, Shift, Zoom, Horizontal Flip
Early Stopping	Enabled
ReduceLROnPlateau	Enabled
Evaluation Metrics	Accuracy, Precision, Recall, F1-score, ROC–AUC,
	Confusion Matrix

**Table 3:** Comparative performance of CNN and transfer learning models.

Method	Accuracy	Precision	Recall	F1-Score	ROC-AUC
CNN	0.8022	0.8004	0.8022	0.7977	0.9369
ResNet50	0.7761	0.8020	0.7761	0.7774	0.9257
DenseNet121	**0.9109**	**0.9151**	**0.9109**	**0.9117**	**0.9844**
MobileNetV2	0.8913	0.8972	0.8913	0.8915	0.9762
EfficientNetB0	0.6174	0.4755	0.6174	0.5346	0.7339

Note: Bold values indicate the highest scores for Accuracy, Precision, Recall, F1-Score, and ROC-AUC among the evaluated models.

## Data Availability

The data presented in this study are openly available in Kaggle at: https://www.kaggle.com/datasets/masoudnickparvar/brain-tumor-mri-dataset (accessed on 12 December 2025). No new data were created during the study.

## Abbreviations

The following abbreviations are used in this manuscript:

DSS	Diagnostic Support Systems
CNN	Convolution Neural Networks
Grad-CAM	Gradient-weighted Class Activation Mapping
LIME	Local Interpretable Model-agnostic Explanations
SHAP	SHapley Additive exPlanations
XAI	Explainable Artificial Intelligence
MRI	Magnetic Resonance Imaging
